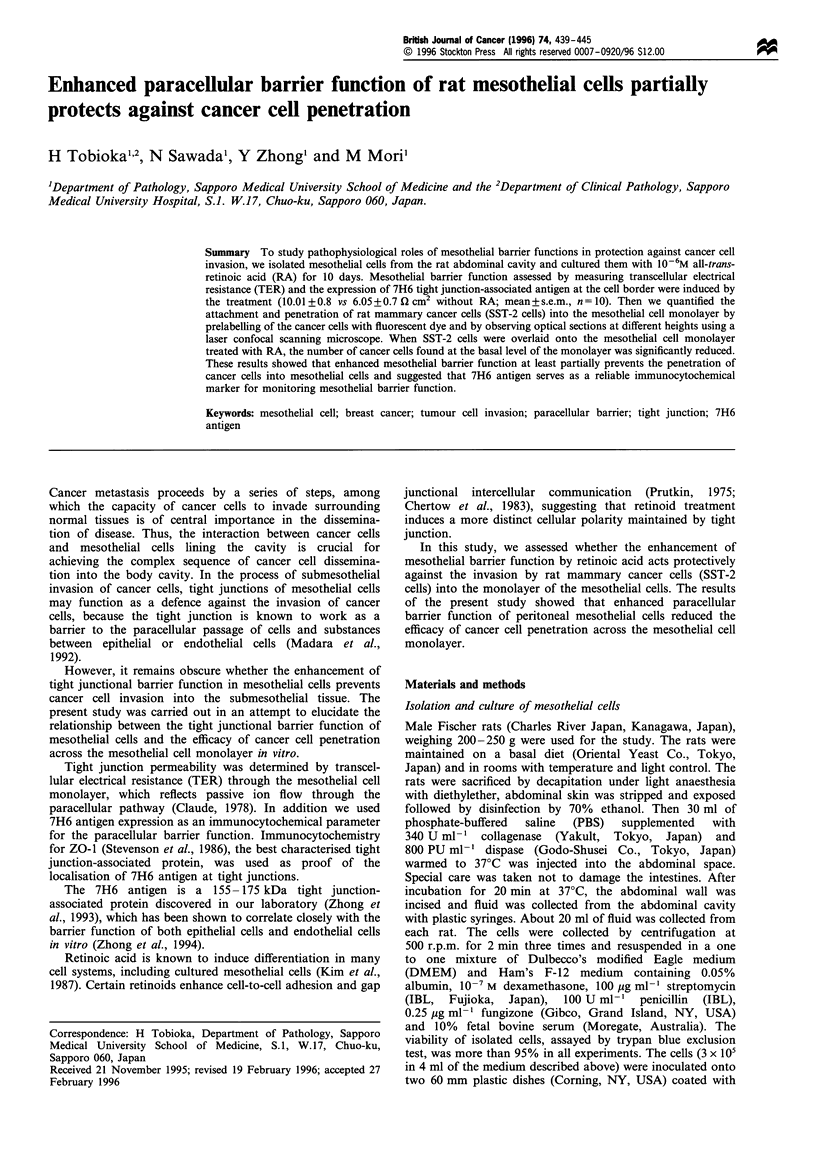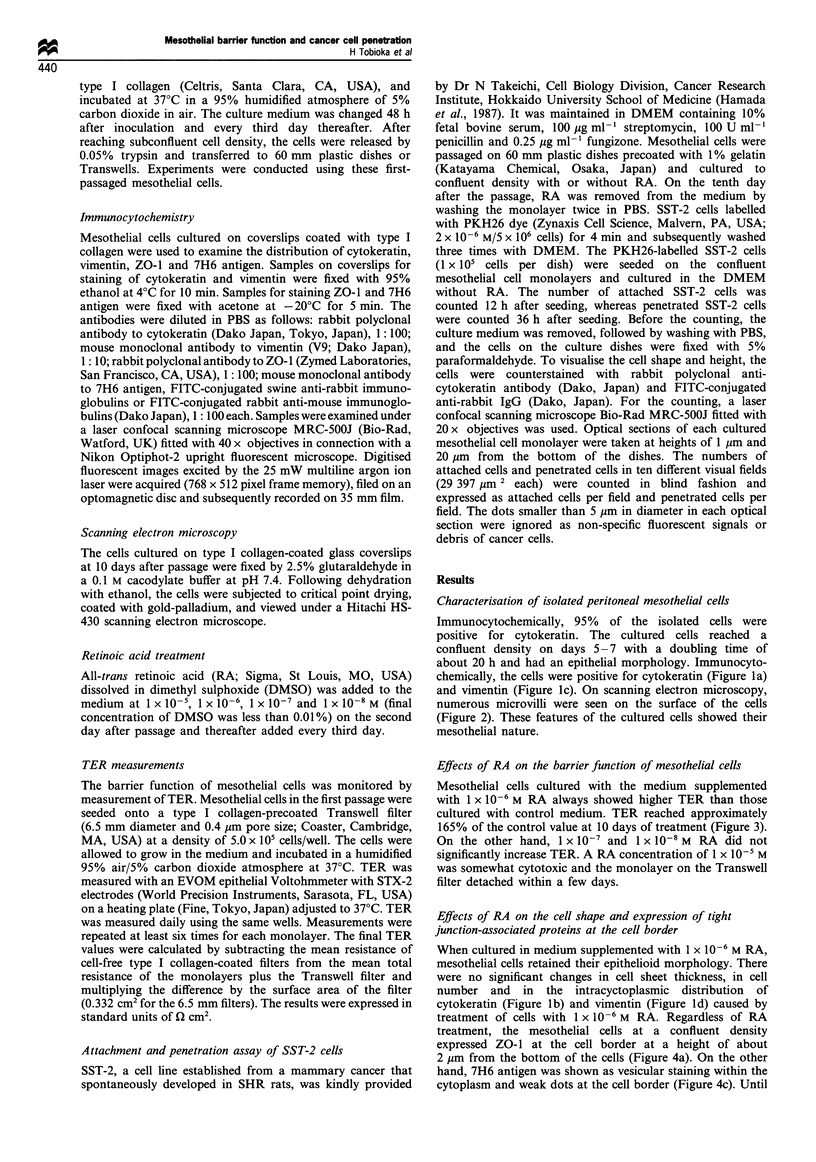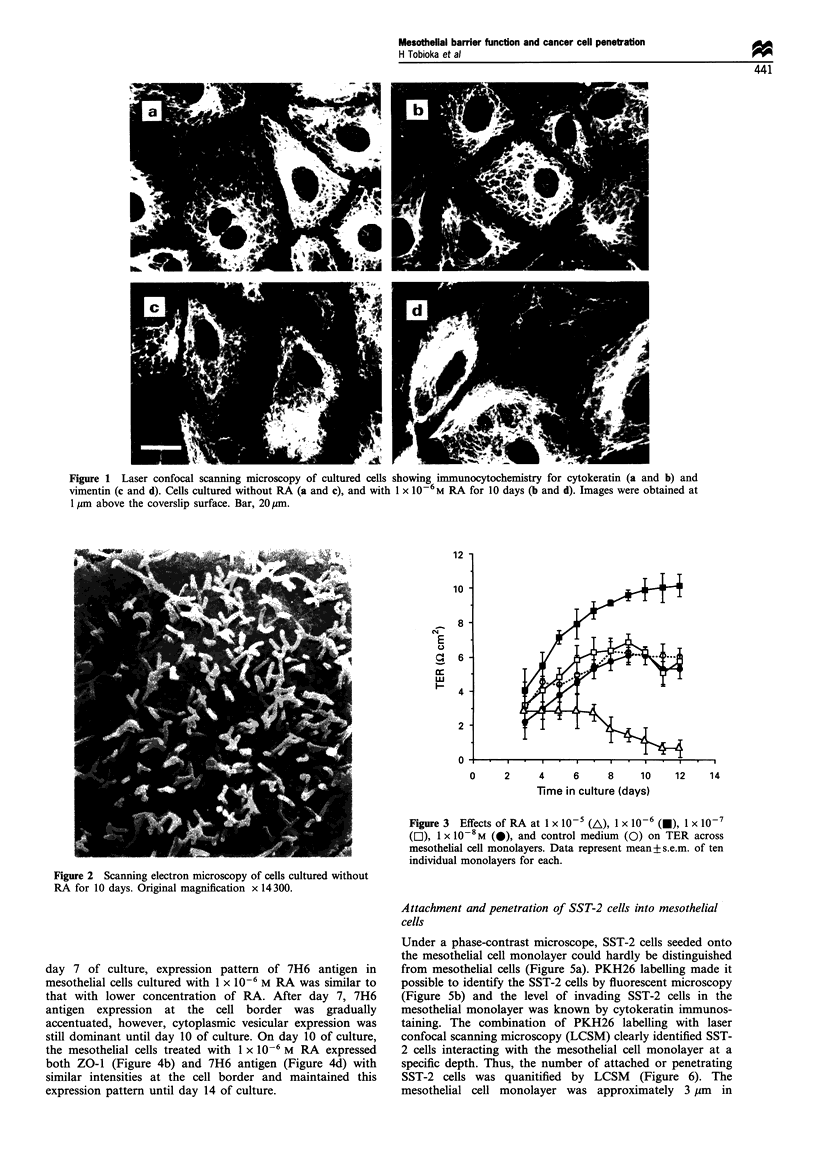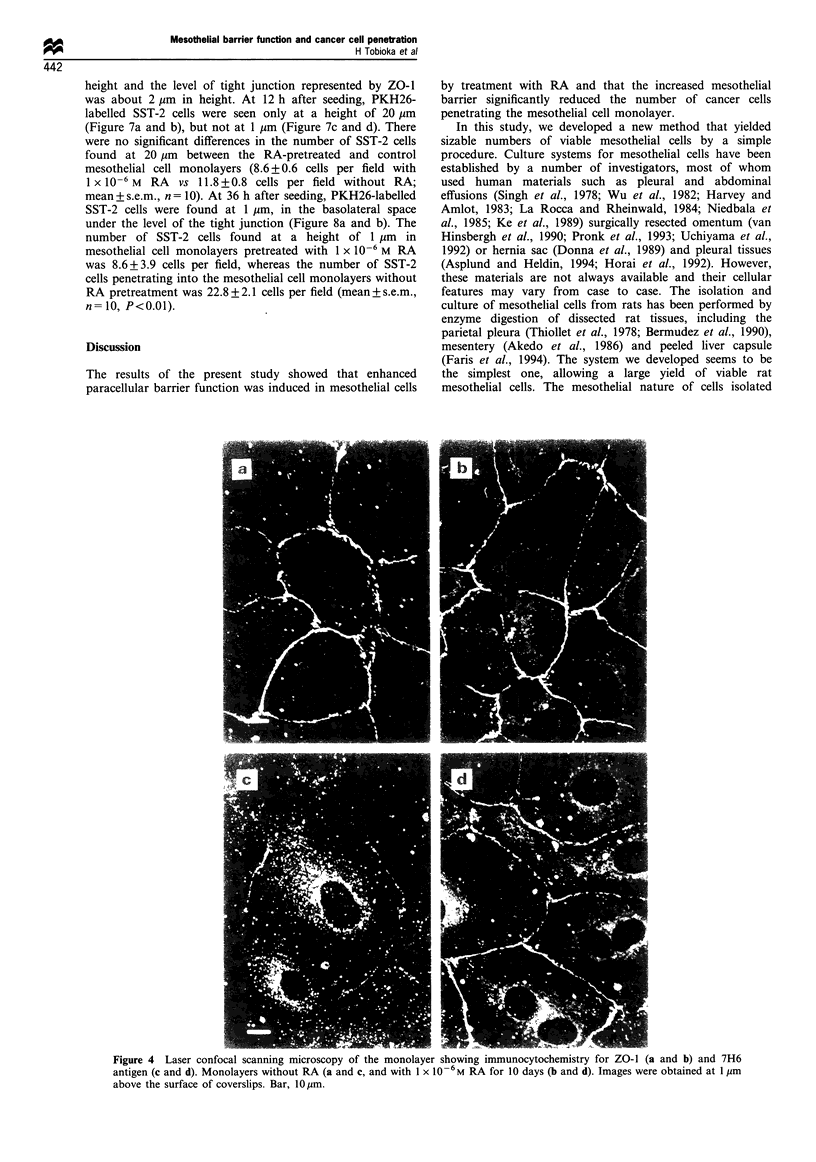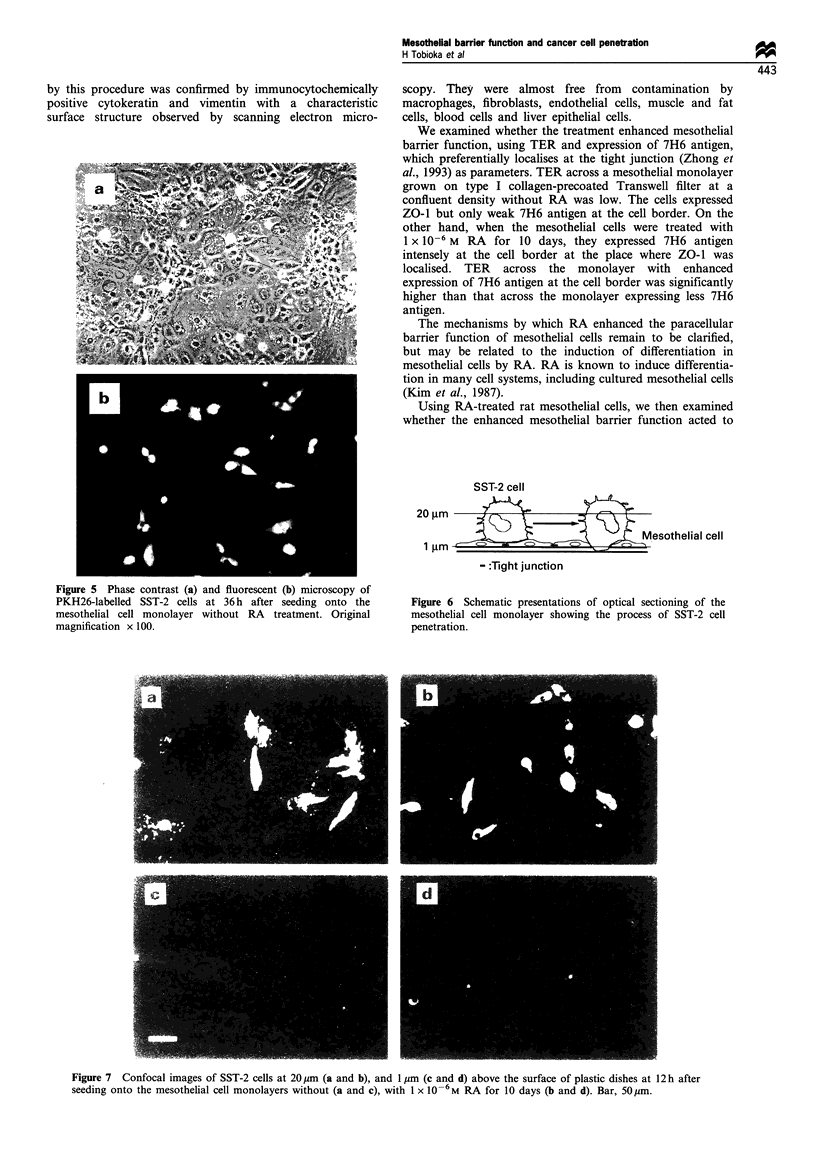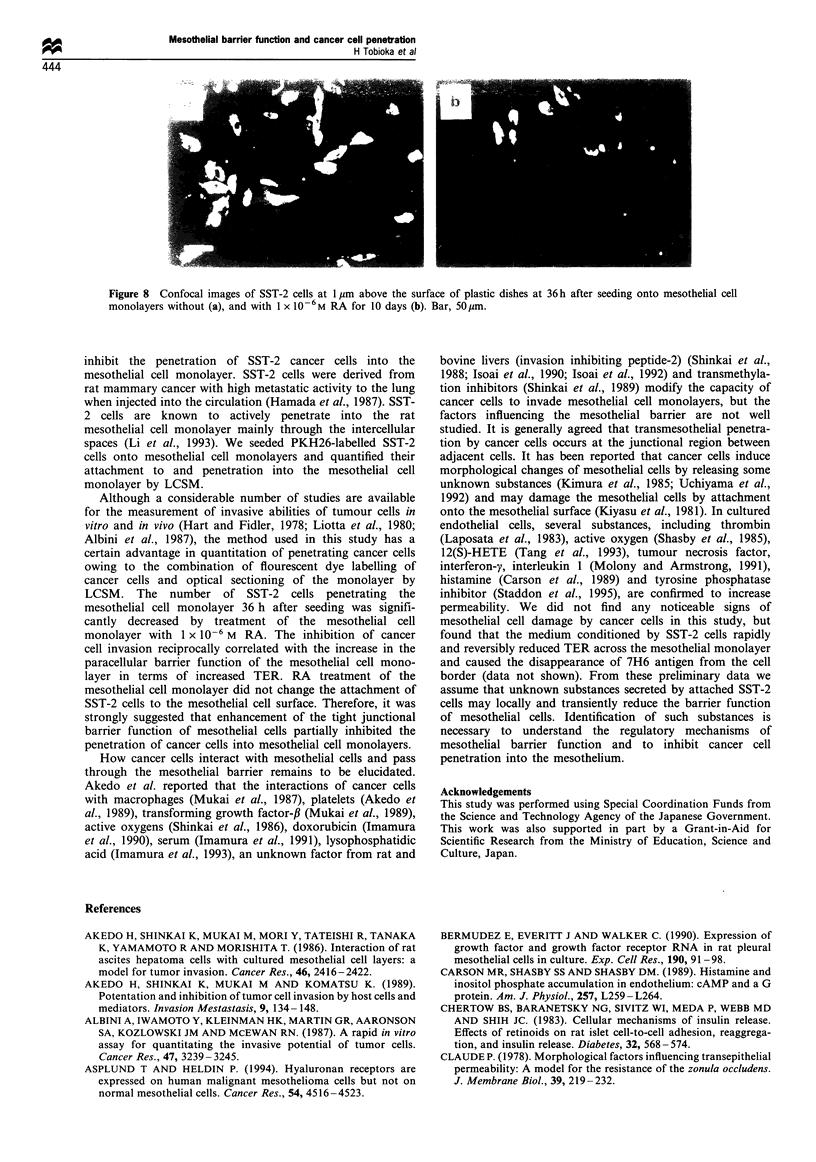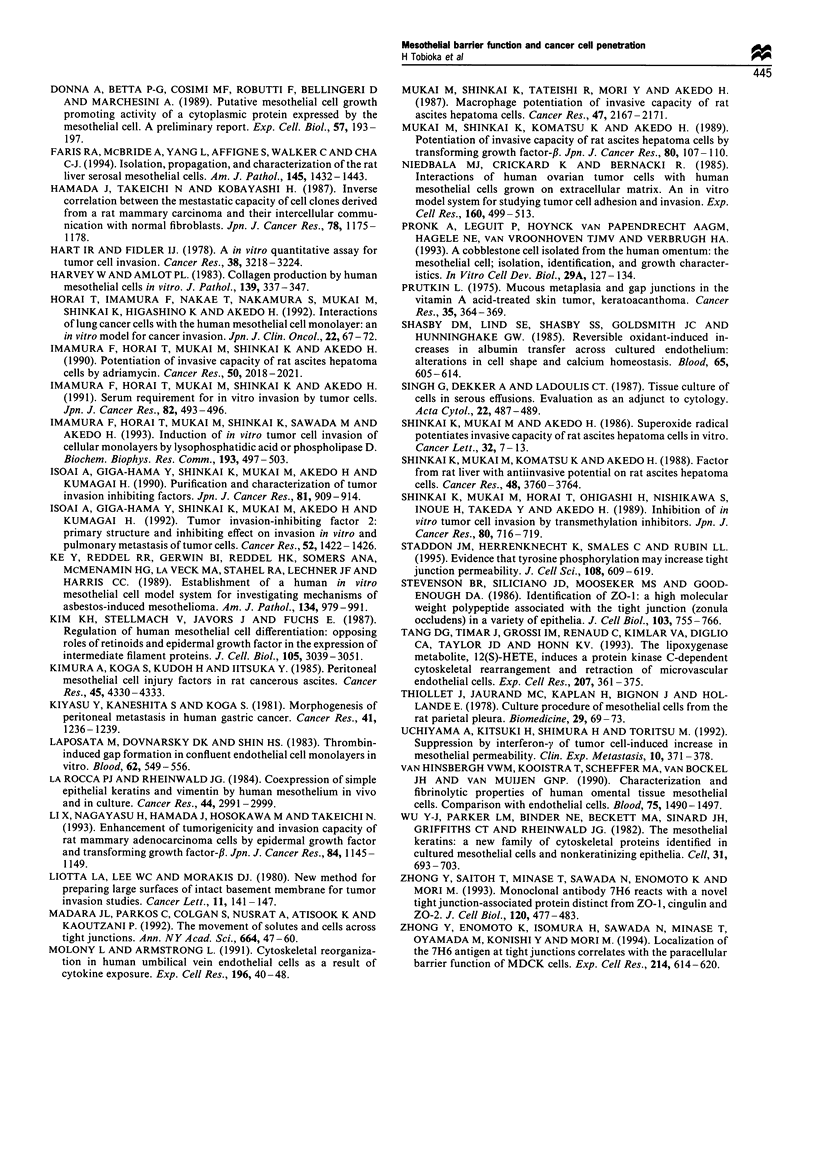# Enhanced paracellular barrier function of rat mesothelial cells partially protects against cancer cell penetration.

**DOI:** 10.1038/bjc.1996.378

**Published:** 1996-08

**Authors:** H. Tobioka, N. Sawada, Y. Zhong, M. Mori

**Affiliations:** Department of Pathology, Sapporo Medical University School of Medicine, Japan.

## Abstract

**Images:**


British Journal of Cancer (1996) 74, 439-445
© 1996 Stockton Press All rights reserved 0007-0920/96 $12.00

### Enhanced paracellular barrier function of rat mesothelial cells partially protects against cancer cell penetration

H Tobioka[1,2], N Sawada[1], Y Zhong[1] and M Mori[1]

[1]Department of Pathology, Sapporo Medical University School of Medicine and the [2]Department of Clinical Pathology, Sapporo Medical University Hospital, S.1. W.17, Chuo-ku, Sapporo 060, Japan.

**Summary** To study pathophysiological roles of mesothelial barrier functions in protection against cancer cell invasion, we isolated mesothelial cells from the rat abdominal cavity and cultured them with $10^{-6}$M all-*trans*-retinoic acid (RA) for 10 days. Mesothelial barrier function assessed by measuring transcellular electrical resistance (TER) and the expression of 7H6 tight junction-associated antigen at the cell border were induced by the treatment ($10.01 \pm 0.8$ *vs* $6.05 \pm 0.7\ \Omega\ cm^2$ without RA; mean±s.e.m., $n = 10$). Then we quantified the attachment and penetration of rat mammary cancer cells (SST-2 cells) into the mesothelial cell monolayer by prelabelling of the cancer cells with fluorescent dye and by observing optical sections at different heights using a laser confocal scanning microscope. When SST-2 cells were overlaid onto the mesothelial cell monolayer treated with RA, the number of cancer cells found at the basal level of the monolayer was significantly reduced. These results showed that enhanced mesothelial barrier function at least partially prevents the penetration of cancer cells into mesothelial cells and suggested that 7H6 antigen serves as a reliable immunocytochemical marker for monitoring mesothelial barrier function.

**Keywords:** mesothelial cell; breast cancer; tumour cell invasion; paracellular barrier; tight junction; 7H6 antigen

Cancer metastasis proceeds by a series of steps, among which the capacity of cancer cells to invade surrounding normal tissues is of central importance in the dissemination of disease. Thus, the interaction between cancer cells and mesothelial cells lining the cavity is crucial for achieving the complex sequence of cancer cell dissemination into the body cavity. In the process of submesothelial invasion of cancer cells, tight junctions of mesothelial cells may function as a defence against the invasion of cancer cells, because the tight junction is known to work as a barrier to the paracellular passage of cells and substances between epithelial or endothelial cells (Madara *et al.*, 1992).

However, it remains obscure whether the enhancement of tight junctional barrier function in mesothelial cells prevents cancer cell invasion into the submesothelial tissue. The present study was carried out in an attempt to elucidate the relationship between the tight junctional barrier function of mesothelial cells and the efficacy of cancer cell penetration across the mesothelial cell monolayer *in vitro*.

Tight junction permeability was determined by transcellular electrical resistance (TER) through the mesothelial cell monolayer, which reflects passive ion flow through the paracellular pathway (Claude, 1978). In addition we used 7H6 antigen expression as an immunocytochemical parameter for the paracellular barrier function. Immunocytochemistry for ZO-1 (Stevenson *et al.*, 1986), the best characterised tight junction-associated protein, was used as proof of the localisation of 7H6 antigen at tight junctions.

The 7H6 antigen is a 155-175 kDa tight junction-associated protein discovered in our laboratory (Zhong *et al.*, 1993), which has been shown to correlate closely with the barrier function of both epithelial cells and endothelial cells *in vitro* (Zhong *et al.*, 1994).

Retinoic acid is known to induce differentiation in many cell systems, including cultured mesothelial cells (Kim *et al.*, 1987). Certain retinoids enhance cell-to-cell adhesion and gap junctional intercellular communication (Prutkin, 1975; Chertow *et al.*, 1983), suggesting that retinoid treatment induces a more distinct cellular polarity maintained by tight junction.

In this study, we assessed whether the enhancement of mesothelial barrier function by retinoic acid acts protectively against the invasion by rat mammary cancer cells (SST-2 cells) into the monolayer of the mesothelial cells. The results of the present study showed that enhanced paracellular barrier function of peritoneal mesothelial cells reduced the efficacy of cancer cell penetration across the mesothelial cell monolayer.

### Materials and methods

### *Isolation and culture of mesothelial cells*

Male Fischer rats (Charles River Japan, Kanagawa, Japan), weighing 200-250 g were used for the study. The rats were maintained on a basal diet (Oriental Yeast Co., Tokyo, Japan) and in rooms with temperature and light control. The rats were sacrificed by decapitation under light anaesthesia with diethylether, abdominal skin was stripped and exposed followed by disinfection by 70% ethanol. Then 30 ml of phosphate-buffered saline (PBS) supplemented with 340 U $ml^{-1}$ collagenase (Yakult, Tokyo, Japan) and 800 PU $ml^{-1}$ dispase (Godo-Shusei Co., Tokyo, Japan) warmed to 37°C was injected into the abdominal space. Special care was taken not to damage the intestines. After incubation for 20 min at 37°C, the abdominal wall was incised and fluid was collected from the abdominal cavity with plastic syringes. About 20 ml of fluid was collected from each rat. The cells were collected by centrifugation at 500 r.p.m. for 2 min three times and resuspended in a one to one mixture of Dulbecco's modified Eagle medium (DMEM) and Ham's F-12 medium containing 0.05% albumin, $10^{-7}$ M dexamethasone, 100 $\mu g\ ml^{-1}$ streptomycin (IBL, Fujioka, Japan), 100 U $ml^{-1}$ penicillin (IBL), 0.25 $\mu g\ ml^{-1}$ fungizone (Gibco, Grand Island, NY, USA) and 10% fetal bovine serum (Moregate, Australia). The viability of isolated cells, assayed by trypan blue exclusion test, was more than 95% in all experiments. The cells ($3 \times 10^5$ in 4 ml of the medium described above) were inoculated onto two 60 mm plastic dishes (Corning, NY, USA) coated with

Correspondence: H Tobioka, Department of Pathology, Sapporo Medical University School of Medicine, S.1, W.17, Chuo-ku, Sapporo 060, Japan
Received 21 November 1995; revised 19 February 1996; accepted 27 February 1996

type I collagen (Celtris, Santa Clara, CA, USA), and incubated at 37°C in a 95% humidified atmosphere of 5% carbon dioxide in air. The culture medium was changed 48 h after inoculation and every third day thereafter. After reaching subconfluent cell density, the cells were released by 0.05% trypsin and transferred to 60 mm plastic dishes or Transwells. Experiments were conducted using these first-passaged mesothelial cells.

*Immunocytochemistry*

Mesothelial cells cultured on coverslips coated with type I collagen were used to examine the distribution of cytokeratin, vimentin, ZO-1 and 7H6 antigen. Samples on coverslips for staining of cytokeratin and vimentin were fixed with 95% ethanol at 4°C for 10 min. Samples for staining ZO-1 and 7H6 antigen were fixed with acetone at −20°C for 5 min. The antibodies were diluted in PBS as follows: rabbit polyclonal antibody to cytokeratin (Dako Japan, Tokyo, Japan), 1 : 100; mouse monoclonal antibody to vimentin (V9; Dako Japan), 1 : 10; rabbit polyclonal antibody to ZO-1 (Zymed Laboratories, San Francisco, CA, USA), 1 : 100; mouse monoclonal antibody to 7H6 antigen, FITC-conjugated swine anti-rabbit immunoglobulins or FITC-conjugated rabbit anti-mouse immunoglobulins (Dako Japan), 1 : 100 each. Samples were examined under a laser confocal scanning microscope MRC-500J (Bio-Rad, Watford, UK) fitted with 40 × objectives in connection with a Nikon Optiphot-2 upright fluorescent microscope. Digitised fluorescent images excited by the 25 mW multiline argon ion laser were acquired (768 × 512 pixel frame memory), filed on an optomagnetic disc and subsequently recorded on 35 mm film.

*Scanning electron microscopy*

The cells cultured on type I collagen-coated glass coverslips at 10 days after passage were fixed by 2.5% glutaraldehyde in a 0.1 M cacodylate buffer at pH 7.4. Following dehydration with ethanol, the cells were subjected to critical point drying, coated with gold-palladium, and viewed under a Hitachi HS-430 scanning electron microscope.

*Retinoic acid treatment*

All-*trans* retinoic acid (RA; Sigma, St Louis, MO, USA) dissolved in dimethyl sulphoxide (DMSO) was added to the medium at $1 \times 10^{-5}$, $1 \times 10^{-6}$, $1 \times 10^{-7}$ and $1 \times 10^{-8}$ M (final concentration of DMSO was less than 0.01%) on the second day after passage and thereafter added every third day.

*TER measurements*

The barrier function of mesothelial cells was monitored by measurement of TER. Mesothelial cells in the first passage were seeded onto a type I collagen-precoated Transwell filter (6.5 mm diameter and 0.4 μm pore size; Coaster, Cambridge, MA, USA) at a density of $5.0 \times 10^5$ cells/well. The cells were allowed to grow in the medium and incubated in a humidified 95% air/5% carbon dioxide atmosphere at 37°C. TER was measured with an EVOM epithelial Voltohmmeter with STX-2 electrodes (World Precision Instruments, Sarasota, FL, USA) on a heating plate (Fine, Tokyo, Japan) adjusted to 37°C. TER was measured daily using the same wells. Measurements were repeated at least six times for each monolayer. The final TER values were calculated by subtracting the mean resistance of cell-free type I collagen-coated filters from the mean total resistance of the monolayers plus the Transwell filter and multiplying the difference by the surface area of the filter (0.332 $cm^2$ for the 6.5 mm filters). The results were expressed in standard units of Ω $cm^2$.

*Attachment and penetration assay of SST-2 cells*

SST-2, a cell line established from a mammary cancer that spontaneously developed in SHR rats, was kindly provided by Dr N Takeichi, Cell Biology Division, Cancer Research Institute, Hokkaido University School of Medicine (Hamada *et al.*, 1987). It was maintained in DMEM containing 10% fetal bovine serum, 100 μg $ml^{-1}$ streptomycin, 100 U $ml^{-1}$ penicillin and 0.25 μg $ml^{-1}$ fungizone. Mesothelial cells were passaged on 60 mm plastic dishes precoated with 1% gelatin (Katayama Chemical, Osaka, Japan) and cultured to confluent density with or without RA. On the tenth day after the passage, RA was removed from the medium by washing the monolayer twice in PBS. SST-2 cells labelled with PKH26 dye (Zynaxis Cell Science, Malvern, PA, USA; $2 \times 10^{-6}$ M/$5 \times 10^6$ cells) for 4 min and subsequently washed three times with DMEM. The PKH26-labelled SST-2 cells ($1 \times 10^5$ cells per dish) were seeded on the confluent mesothelial cell monolayers and cultured in the DMEM without RA. The number of attached SST-2 cells was counted 12 h after seeding, whereas penetrated SST-2 cells were counted 36 h after seeding. Before the counting, the culture medium was removed, followed by washing with PBS, and the cells on the culture dishes were fixed with 5% paraformaldehyde. To visualise the cell shape and height, the cells were counterstained with rabbit polyclonal anti-cytokeratin antibody (Dako, Japan) and FITC-conjugated anti-rabbit IgG (Dako, Japan). For the counting, a laser confocal scanning microscope Bio-Rad MRC-500J fitted with 20 × objectives was used. Optical sections of each cultured mesothelial cell monolayer were taken at heights of 1 μm and 20 μm from the bottom of the dishes. The numbers of attached cells and penetrated cells in ten different visual fields (29 397 $\mu m^2$ each) were counted in blind fashion and expressed as attached cells per field and penetrated cells per field. The dots smaller than 5 μm in diameter in each optical section were ignored as non-specific fluorescent signals or debris of cancer cells.

### Results

*Characterisation of isolated peritoneal mesothelial cells*

Immunocytochemically, 95% of the isolated cells were positive for cytokeratin. The cultured cells reached a confluent density on days 5–7 with a doubling time of about 20 h and had an epithelial morphology. Immunocytochemically, the cells were positive for cytokeratin (Figure 1a) and vimentin (Figure 1c). On scanning electron microscopy, numerous microvilli were seen on the surface of the cells (Figure 2). These features of the cultured cells showed their mesothelial nature.

*Effects of RA on the barrier function of mesothelial cells*

Mesothelial cells cultured with the medium supplemented with $1 \times 10^{-6}$ M RA always showed higher TER than those cultured with control medium. TER reached approximately 165% of the control value at 10 days of treatment (Figure 3). On the other hand, $1 \times 10^{-7}$ and $1 \times 10^{-8}$ M RA did not significantly increase TER. A RA concentration of $1 \times 10^{-5}$ M was somewhat cytotoxic and the monolayer on the Transwell filter detached within a few days.

*Effects of RA on the cell shape and expression of tight junction-associated proteins at the cell border*

When cultured in medium supplemented with $1 \times 10^{-6}$ M RA, mesothelial cells retained their epithelioid morphology. There were no significant changes in cell sheet thickness, in cell number and in the intracyctoplasmic distribution of cytokeratin (Figure 1b) and vimentin (Figure 1d) caused by treatment of cells with $1 \times 10^{-6}$ M RA. Regardless of RA treatment, the mesothelial cells at a confluent density expressed ZO-1 at the cell border at a height of about 2 μm from the bottom of the cells (Figure 4a). On the other hand, 7H6 antigen was shown as vesicular staining within the cytoplasm and weak dots at the cell border (Figure 4c). Until

**Figure 1** Laser confocal scanning microscopy of cultured cells showing immunocytochemistry for cytokeratin (**a** and **b**) and vimentin (**c** and **d**). Cells cultured without RA (**a** and **c**), and with $1 \times 10^{-6}$ M RA for 10 days (**b** and **d**). Images were obtained at 1 $\mu$m above the coverslip surface. Bar, 20 $\mu$m.

**Figure 2** Scanning electron microscopy of cells cultured without RA for 10 days. Original magnification ×14 300.

**Figure 3** Effects of RA at $1 \times 10^{-5}$ (△), $1 \times 10^{-6}$ (■), $1 \times 10^{-7}$ (□), $1 \times 10^{-8}$ M (●), and control medium (○) on TER across mesothelial cell monolayers. Data represent mean ± s.e.m. of ten individual monolayers for each.

day 7 of culture, expression pattern of 7H6 antigen in mesothelial cells cultured with $1 \times 10^{-6}$ M RA was similar to that with lower concentration of RA. After day 7, 7H6 antigen expression at the cell border was gradually accentuated, however, cytoplasmic vesicular expression was still dominant until day 10 of culture. On day 10 of culture, the mesothelial cells treated with $1 \times 10^{-6}$ M RA expressed both ZO-1 (Figure 4b) and 7H6 antigen (Figure 4d) with similar intensities at the cell border and maintained this expression pattern until day 14 of culture.

*Attachment and penetration of SST-2 cells into mesothelial cells*

Under a phase-contrast microscope, SST-2 cells seeded onto the mesothelial cell monolayer could hardly be distinguished from mesothelial cells (Figure 5a). PKH26 labelling made it possible to identify the SST-2 cells by fluorescent microscopy (Figure 5b) and the level of invading SST-2 cells in the mesothelial monolayer was known by cytokeratin immunostaining. The combination of PKH26 labelling with laser confocal scanning microscopy (LCSM) clearly identified SST-2 cells interacting with the mesothelial cell monolayer at a specific depth. Thus, the number of attached or penetrating SST-2 cells was quanitified by LCSM (Figure 6). The mesothelial cell monolayer was approximately 3 $\mu$m in height and the level of tight junction represented by ZO-1 was about 2 $\mu$m in height. At 12 h after seeding, PKH26-labelled SST-2 cells were seen only at a height of 20 $\mu$m (Figure 7a and b), but not at 1 $\mu$m (Figure 7c and d). There were no significant differences in the number of SST-2 cells found at 20 $\mu$m between the RA-pretreated and control mesothelial cell monolayers (8.6$\pm$0.6 cells per field with $1\times10^{-6}$ M RA *vs* 11.8$\pm$0.8 cells per field without RA; mean$\pm$s.e.m., $n=10$). At 36 h after seeding, PKH26-labelled SST-2 cells were found at 1 $\mu$m, in the basolateral space under the level of the tight junction (Figure 8a and b). The number of SST-2 cells found at a height of 1 $\mu$m in mesothelial cell monolayers pretreated with $1\times10^{-6}$ M RA was 8.6$\pm$3.9 cells per field, whereas the number of SST-2 cells penetrating into the mesothelial cell monolayers without RA pretreatment was 22.8$\pm$2.1 cells per field (mean$\pm$s.e.m., $n=10$, $P<0.01$).

### Discussion

The results of the present study showed that enhanced paracellular barrier function was induced in mesothelial cells by treatment with RA and that the increased mesothelial barrier significantly reduced the number of cancer cells penetrating the mesothelial cell monolayer.

In this study, we developed a new method that yielded sizable numbers of viable mesothelial cells by a simple procedure. Culture systems for mesothelial cells have been established by a number of investigators, most of whom used human materials such as pleural and abdominal effusions (Singh *et al.*, 1978; Wu *et al.*, 1982; Harvey and Amlot, 1983; La Rocca and Rheinwald, 1984; Niedbala *et al.*, 1985; Ke *et al.*, 1989) surgically resected omentum (van Hinsbergh *et al.*, 1990; Pronk *et al.*, 1993; Uchiyama *et al.*, 1992) or hernia sac (Donna *et al.*, 1989) and pleural tissues (Asplund and Heldin, 1994; Horai *et al.*, 1992). However, these materials are not always available and their cellular features may vary from case to case. The isolation and culture of mesothelial cells from rats has been performed by enzyme digestion of dissected rat tissues, including the parietal pleura (Thiollet *et al.*, 1978; Bermudez *et al.*, 1990), mesentery (Akedo *et al.*, 1986) and peeled liver capsule (Faris *et al.*, 1994). The system we developed seems to be the simplest one, allowing a large yield of viable rat mesothelial cells. The mesothelial nature of cells isolated

**Figure 4** Laser confocal scanning microscopy of the monolayer showing immunocytochemistry for ZO-1 (**a** and **b**) and 7H6 antigen (**c** and **d**). Monolayers without RA (**a** and **c**, and with $1\times10^{-6}$ M RA for 10 days (**b** and **d**). Images were obtained at 1 $\mu$m above the surface of coverslips. Bar, 10 $\mu$m.

by this procedure was confirmed by immunocytochemically positive cytokeratin and vimentin with a characteristic surface structure observed by scanning electron microscopy. They were almost free from contamination by macrophages, fibroblasts, endothelial cells, muscle and fat cells, blood cells and liver epithelial cells.

We examined whether the treatment enhanced mesothelial barrier function, using TER and expression of 7H6 antigen, which preferentially localises at the tight junction (Zhong *et al.*, 1993) as parameters. TER across a mesothelial monolayer grown on type I collagen-precoated Transwell filter at a confluent density without RA was low. The cells expressed ZO-1 but only weak 7H6 antigen at the cell border. On the other hand, when the mesothelial cells were treated with $1 \times 10^{-6}$ M RA for 10 days, they expressed 7H6 antigen intensely at the cell border at the place where ZO-1 was localised. TER across the monolayer with enhanced expression of 7H6 antigen at the cell border was significantly higher than that across the monolayer expressing less 7H6 antigen.

The mechanisms by which RA enhanced the paracellular barrier function of mesothelial cells remain to be clarified, but may be related to the induction of differentiation in mesothelial cells by RA. RA is known to induce differentiation in many cell systems, including cultured mesothelial cells (Kim *et al.*, 1987).

Using RA-treated rat mesothelial cells, we then examined whether the enhanced mesothelial barrier function acted to

**Figure 5** Phase contrast (**a**) and fluorescent (**b**) microscopy of PKH26-labelled SST-2 cells at 36 h after seeding onto the mesothelial cell monolayer without RA treatment. Original magnification ×100.

**Figure 6** Schematic presentations of optical sectioning of the mesothelial cell monolayer showing the process of SST-2 cell penetration.

**Figure 7** Confocal images of SST-2 cells at 20 $\mu$m (**a** and **b**), and 1 $\mu$m (**c** and **d**) above the surface of plastic dishes at 12 h after seeding onto the mesothelial cell monolayers without (**a** and **c**), with $1 \times 10^{-6}$ M RA for 10 days (**b** and **d**). Bar, 50 $\mu$m.

**Figure 8** Confocal images of SST-2 cells at 1 $\mu$m above the surface of plastic dishes at 36 h after seeding onto mesothelial cell monolayers without (**a**), and with $1 \times 10^{-6}$ M RA for 10 days (**b**). Bar, 50 $\mu$m.

inhibit the penetration of SST-2 cancer cells into the mesothelial cell monolayer. SST-2 cells were derived from rat mammary cancer with high metastatic activity to the lung when injected into the circulation (Hamada *et al.*, 1987). SST-2 cells are known to actively penetrate into the rat mesothelial cell monolayer mainly through the intercellular spaces (Li *et al.*, 1993). We seeded PKH26-labelled SST-2 cells onto mesothelial cell monolayers and quantified their attachment to and penetration into the mesothelial cell monolayer by LCSM.

Although a considerable number of studies are available for the measurement of invasive abilities of tumour cells *in vitro* and *in vivo* (Hart and Fidler, 1978; Liotta *et al.*, 1980; Albini *et al.*, 1987), the method used in this study has a certain advantage in quantitation of penetrating cancer cells owing to the combination of flourescent dye labelling of cancer cells and optical sectioning of the monolayer by LCSM. The number of SST-2 cells penetrating the mesothelial cell monolayer 36 h after seeding was significantly decreased by treatment of the mesothelial cell monolayer with $1 \times 10^{-6}$ M RA. The inhibition of cancer cell invasion reciprocally correlated with the increase in the paracellular barrier function of the mesothelial cell monolayer in terms of increased TER. RA treatment of the mesothelial cell monolayer did not change the attachment of SST-2 cells to the mesothelial cell surface. Therefore, it was strongly suggested that enhancement of the tight junctional barrier function of mesothelial cells partially inhibited the penetration of cancer cells into mesothelial cell monolayers.

How cancer cells interact with mesothelial cells and pass through the mesothelial barrier remains to be elucidated. Akedo *et al.* reported that the interactions of cancer cells with macrophages (Mukai *et al.*, 1987), platelets (Akedo *et al.*, 1989), transforming growth factor-$\beta$ (Mukai *et al.*, 1989), active oxygens (Shinkai *et al.*, 1986), doxorubicin (Imamura *et al.*, 1990), serum (Imamura *et al.*, 1991), lysophosphatidic acid (Imamura *et al.*, 1993), an unknown factor from rat and bovine livers (invasion inhibiting peptide-2) (Shinkai *et al.*, 1988; Isoai *et al.*, 1990; Isoai *et al.*, 1992) and transmethylation inhibitors (Shinkai *et al.*, 1989) modify the capacity of cancer cells to invade mesothelial cell monolayers, but the factors influencing the mesothelial barrier are not well studied. It is generally agreed that transmesothelial penetration by cancer cells occurs at the junctional region between adjacent cells. It has been reported that cancer cells induce morphological changes of mesothelial cells by releasing some unknown substances (Kimura *et al.*, 1985; Uchiyama *et al.*, 1992) and may damage the mesothelial cells by attachment onto the mesothelial surface (Kiyasu *et al.*, 1981). In cultured endothelial cells, several substances, including thrombin (Laposata *et al.*, 1983), active oxygen (Shasby *et al.*, 1985), 12(S)-HETE (Tang *et al.*, 1993), tumour necrosis factor, interferon-$\gamma$, interleukin 1 (Molony and Armstrong, 1991), histamine (Carson *et al.*, 1989) and tyrosine phosphatase inhibitor (Staddon *et al.*, 1995), are confirmed to increase permeability. We did not find any noticeable signs of mesothelial cell damage by cancer cells in this study, but found that the medium conditioned by SST-2 cells rapidly and reversibly reduced TER across the mesothelial monolayer and caused the disappearance of 7H6 antigen from the cell border (data not shown). From these preliminary data we assume that unknown substances secreted by attached SST-2 cells may locally and transiently reduce the barrier function of mesothelial cells. Identification of such substances is necessary to understand the regulatory mechanisms of mesothelial barrier function and to inhibit cancer cell penetration into the mesothelium.

**Acknowledgements**
This study was performed using Special Coordination Funds from the Science and Technology Agency of the Japanese Government. This work was also supported in part by a Grant-in-Aid for Scientific Research from the Ministry of Education, Science and Culture, Japan.

### References

AKEDO H, SHINKAI K, MUKAI M, MORI Y, TATEISHI R, TANAKA K, YAMAMOTO R AND MORISHITA T. (1986). Interaction of rat ascites hepatoma cells with cultured mesothelial cell layers: a model for tumor invasion. *Cancer Res.*, **46**, 2416–2422.

AKEDO H, SHINKAI K, MUKAI M AND KOMATSU K. (1989). Potentation and inhibition of tumor cell invasion by host cells and mediators. *Invasion Mestastasis*, **9**, 134–148.

ALBINI A, IWAMOTO Y, KLEINMAN HK, MARTIN GR, AARONSON SA, KOZLOWSKI JM AND MCEWAN RN. (1987). A rapid *in vitro* assay for quantitating the invasive potential of tumor cells. *Cancer Res.*, **47**, 3239–3245.

ASPLUND T AND HELDIN P. (1994). Hyaluronan receptors are expressed on human malignant mesothelioma cells but not on normal mesothelial cells. *Cancer Res.*, **54**, 4516–4523.

BERMUDEZ E, EVERITT J AND WALKER C. (1990). Expression of growth factor and growth factor receptor RNA in rat pleural mesothelial cells in culture. *Exp. Cell Res.*, **190**, 91–98.

CARSON MR, SHASBY SS AND SHASBY DM. (1989). Histamine and inositol phosphate accumulation in endothelium: cAMP and a G protein. *Am. J. Physiol.*, **257**, L259–L264.

CHERTOW BS, BARANETSKY NG, SIVITZ WI, MEDA P, WEBB MD AND SHIH JC. (1983). Cellular mechanisms of insulin release. Effects of retinoids on rat islet cell-to-cell adhesion, reaggregation, and insulin release. *Diabetes*, **32**, 568–574.

CLAUDE P. (1978). Morphological factors influencing transepithelial permeability: A model for the resistance of the *zonula occludens*. *J. Membrane Biol.*, **39**, 219–232.

DONNA A, BETTA P-G, COSIMI MF, ROBUTTI F, BELLINGERI D AND MARCHESINI A. (1989). Putative mesothelial cell growth promoting activity of a cytoplasmic protein expressed by the mesothelial cell. A preliminary report. *Exp. Cell. Biol.*, **57**, 193–197.

FARIS RA, MCBRIDE A, YANG L, AFFIGNE S, WALKER C AND CHA C-J. (1994). Isolation, propagation, and characterization of the rat liver serosal mesothelial cells. *Am. J. Pathol.*, **145**, 1432–1443.

HAMADA J, TAKEICHI N AND KOBAYASHI H. (1987). Inverse correlation between the mestastatic capacity of cell clones derived from a rat mammary carcinoma and their intercellular communication with normal fibroblasts. *Jpn. J. Cancer Res.*, **78**, 1175–1178.

HART IR AND FIDLER IJ. (1978). A *in vitro* quantitative assay for tumor cell invasion. *Cancer Res.*, **38**, 3218–3224.

HARVEY W AND AMLOT PL. (1983). Collagen production by human mesothelial cells *in vitro*. *J. Pathol.*, **139**, 337–347.

HORAI T, IMAMURA F, NAKAE T, NAKAMURA S, MUKAI M, SHINKAI K, HIGASHINO K AND AKEDO H. (1992). Interactions of lung cancer cells with the human mesothelial cell monolayer: an *in vitro* model for cancer invasion. *Jpn. J. Clin. Oncol.*, **22**, 67–72.

IMAMURA F, HORAI T, MUKAI M, SHINKAI K AND AKEDO H. (1990). Potentiation of invasive capacity of rat ascites hepatoma cells by adriamycin. *Cancer Res.*, **50**, 2018–2021.

IMAMURA F, HORAI T, MUKAI M, SHINKAI K AND AKEDO H. (1991). Serum requirement for in vitro invasion by tumor cells. *Jpn. J. Cancer Res.*, **82**, 493–496.

IMAMURA F, HORAI T, MUKAI M, SHINKAI K, SAWADA M AND AKEDO H. (1993). Induction of *in vitro* tumor cell invasion of cellular monolayers by lysophosphatidic acid or phospholipase D. *Biochem. Biophys. Res. Comm.*, **193**, 497–503.

ISOAI A, GIGA-HAMA Y, SHINKAI K, MUKAI M, AKEDO H AND KUMAGAI H. (1990). Purification and characterization of tumor invasion inhibiting factors. *Jpn. J. Cancer Res.*, **81**, 909–914.

ISOAI A, GIGA-HAMA Y, SHINKAI K, MUKAI M, AKEDO H AND KUMAGAI H. (1992). Tumor invasion-inhibiting factor 2: primary structure and inhibiting effect on invasion *in vitro* and pulmonary metastasis of tumor cells. *Cancer Res.*, **52**, 1422–1426.

KE Y, REDDEL RR, GERWIN BI, REDDEL HK, SOMERS ANA, MCMENAMIN HG, LA VECK MA, STAHEL RA, LECHNER JF AND HARRIS CC. (1989). Establishment of a human *in vitro* mesothelial cell model system for investigating mechanisms of asbestos-induced mesothelioma. *Am. J. Pathol.*, **134**, 979–991.

KIM KH, STELLMACH V, JAVORS J AND FUCHS E. (1987). Regulation of human mesothelial cell differentiation: opposing roles of retinoids and epidermal growth factor in the expression of intermediate filament proteins. *J. Cell. Biol.*, **105**, 3039–3051.

KIMURA A, KOGA S, KUDOH H AND IITSUKA Y. (1985). Peritoneal mesothelial cell injury factors in rat cancerous ascites. *Cancer Res.*, **45**, 4330–4333.

KIYASU Y, KANESHITA S AND KOGA S. (1981). Morphogenesis of peritoneal metastasis in human gastric cancer. *Cancer Res.*, **41**, 1236–1239.

LAPOSATA M, DOVNARSKY DK AND SHIN HS. (1983). Thrombin-induced gap formation in confluent endothelial cell monolayers in vitro. *Blood*, **62**, 549–556.

LA ROCCA PJ AND RHEINWALD JG. (1984). Coexpression of simple epithelial keratins and vimentin by human mesothelium in vivo and in culture. *Cancer Res.*, **44**, 2991–2999.

LI X, NAGAYASU H, HAMADA J, HOSOKAWA M AND TAKEICHI N. (1993). Enhancement of tumorigenicity and invasion capacity of rat mammary adenocarcinoma cells by epidermal growth factor and transforming growth factor-$\beta$. *Jpn. J. Cancer Res.*, **84**, 1145–1149.

LIOTTA LA, LEE WC AND MORAKIS DJ. (1980). New method for preparing large surfaces of intact basement membrane for tumor invasion studies. *Cancer Lett.*, **11**, 141–147.

MADARA JL, PARKOS C, COLGAN S, NUSRAT A, ATISOOK K AND KAOUTZANI P. (1992). The movement of solutes and cells across tight junctions. *Ann. NY Acad. Sci.*, **664**, 47–60.

MOLONY L AND ARMSTRONG L. (1991). Cytoskeletal reorganization in human umbilical vein endothelial cells as a result of cytokine exposure. *Exp. Cell Res.*, **196**, 40–48.

MUKAI M, SHINKAI K, TATEISHI R, MORI Y AND AKEDO H. (1987). Macrophage potentiation of invasive capacity of rat ascites hepatoma cells. *Cancer Res.*, **47**, 2167–2171.

MUKAI M, SHINKAI K, KOMATSU K AND AKEDO H. (1989). Potentiation of invasive capacity of rat ascites hepatoma cells by transforming growth factor-$\beta$. *Jpn. J. Cancer Res.*, **80**, 107–110.

NIEDBALA MJ, CRICKARD K AND BERNACKI R. (1985). Interactions of human ovarian tumor cells with human mesothelial cells grown on extracellular matrix. An in vitro model system for studying tumor cell adhesion and invasion. *Exp. Cell Res.*, **160**, 499–513.

PRONK A, LEGUIT P, HOYNCK VAN PAPENDRECHT AAGM, HAGELE NE, VAN VROONHOVEN TJMV AND VERBRUGH HA. (1993). A cobblestone cell isolated from the human omentum: the mesothelial cell; isolation, identification, and growth characteristics. *In Vitro Cell Dev. Biol.*, **29A**, 127–134.

PRUTKIN L. (1975). Mucous metaplasia and gap junctions in the vitamin A acid-treated skin tumor, keratoacanthoma. *Cancer Res.*, **35**, 364–369.

SHASBY DM, LIND SE, SHASBY SS, GOLDSMITH JC AND HUNNINGHAKE GW. (1985). Reversible oxidant-induced increases in albumin transfer across cultured endothelium: alterations in cell shape and calcium homeostasis. *Blood*, **65**, 605–614.

SINGH G, DEKKER A AND LADOULIS CT. (1987). Tissue culture of cells in serous effusions. Evaluation as an adjunct to cytology. *Acta Cytol.*, **22**, 487–489.

SHINKAI K, MUKAI M AND AKEDO H. (1986). Superoxide radical potentiates invasive capacity of rat ascites hepatoma cells in vitro. *Cancer Lett.*, **32**, 7–13.

SHINKAI K, MUKAI M, KOMATSU K AND AKEDO H. (1988). Factor from rat liver with antiinvasive potential on rat ascites hepatoma cells. *Cancer Res.*, **48**, 3760–3764.

SHINKAI K, MUKAI M, HORAI T, OHIGASHI H, NISHIKAWA S, INOUE H, TAKEDA Y AND AKEDO H. (1989). Inhibition of *in vitro* tumor cell invasion by transmethylation inhibitors. *Jpn. J. Cancer Res.*, **80**, 716–719.

STADDON JM, HERRENKNECHT K, SMALES C AND RUBIN LL. (1995). Evidence that tyrosine phosphorylation may increase tight junction permeability. *J. Cell Sci.*, **108**, 609–619.

STEVENSON BR, SILICIANO JD, MOOSEKER MS AND GOODENOUGH DA. (1986). Identification of ZO-1: a high molecular weight polypeptide associated with the tight junction (zonula occludens) in a variety of epithelia. *J. Cell Biol.*, **103**, 755–766.

TANG DG, TIMAR J, GROSSI IM, RENAUD C, KIMLAR VA, DIGLIO CA, TAYLOR JD AND HONN KV. (1993). The lipoxygenase metabolite, 12(S)-HETE, induces a protein kinase C-dependent cytoskeletal rearrangement and retraction of microvascular endothelial cells. *Exp. Cell Res.*, **207**, 361–375.

THIOLLET J, JAURAND MC, KAPLAN H, BIGNON J AND HOLLANDE E. (1978). Culture procedure of mesothelial cells from the rat parietal pleura. *Biomedicine*, **29**, 69–73.

UCHIYAMA A, KITSUKI H, SHIMURA H AND TORITSU M. (1992). Suppression by interferon-$\gamma$ of tumor cell-induced increase in mesothelial permeability. *Clin. Exp. Metastasis*, **10**, 371–378.

VAN HINSBERGH VWM, KOOISTRA T, SCHEFFER MA, VAN BOCKEL JH AND VAN MUIJEN GNP. (1990). Characterization and fibrinolytic properties of human omental tissue mesothelial cells. Comparison with endothelial cells. *Blood*, **75**, 1490–1497.

WU Y-J, PARKER LM, BINDER NE, BECKETT MA, SINARD JH, GRIFFITHS CT AND RHEINWALD JG. (1982). The mesothelial keratins: a new family of cytoskeletal proteins identified in cultured mesothelial cells and nonkeratinizing epithelia. *Cell*, **31**, 693–703.

ZHONG Y, SAITOH T, MINASE T, SAWADA N, ENOMOTO K AND MORI M. (1993). Monoclonal antibody 7H6 reacts with a novel tight junction-associated protein distinct from ZO-1, cingulin and ZO-2. *J. Cell Biol.*, **120**, 477–483.

ZHONG Y, ENOMOTO K, ISOMURA H, SAWADA N, MINASE T, OYAMADA M, KONISHI Y AND MORI M. (1994). Localization of the 7H6 antigen at tight junctions correlates with the paracellular barrier function of MDCK cells. *Exp. Cell Res.*, **214**, 614–620.